# Global mosquito observations dashboard (GMOD): creating a user-friendly web interface fueled by citizen science to monitor invasive and vector mosquitoes

**DOI:** 10.1186/s12942-023-00350-7

**Published:** 2023-10-28

**Authors:** Johnny A. Uelmen, Andrew Clark, John Palmer, Jared Kohler, Landon C. Van Dyke, Russanne Low, Connor D. Mapes, Ryan M. Carney

**Affiliations:** 1 Department of Integrative Biology, University of South Florida (USF), Tampa, FL 33620 USA; 2 Institute for Global Environmental Strategies, Arlington, VA 22202 USA; 3 Department of Political and Social Sciences, Universitat Pompeau Fabra, 08005 Barcelona, Spain; 4 Esri, Redlands, CA 92373 USA; 5 United States Department of State, Washington, DC 20520 USA; 6 Department of Geography, University of Glasgow, Glasgow, G12 8QQ Scotland, UK

**Keywords:** *Anopheles stephensi*, Citizen science, Data, Dashboard, GIS, Malaria, Mosquito, Open source, Surveillance, Vector-borne disease

## Abstract

**Background:**

Mosquitoes and the diseases they transmit pose a significant public health threat worldwide, causing more fatalities than any other animal. To effectively combat this issue, there is a need for increased public awareness and mosquito control. However, traditional surveillance programs are time-consuming, expensive, and lack scalability. Fortunately, the widespread availability of mobile devices with high-resolution cameras presents a unique opportunity for mosquito surveillance. In response to this, the Global Mosquito Observations Dashboard (GMOD) was developed as a free, public platform to improve the detection and monitoring of invasive and vector mosquitoes through citizen science participation worldwide.

**Methods:**

GMOD is an interactive web interface that collects and displays mosquito observation and habitat data supplied by four datastreams with data generated by citizen scientists worldwide. By providing information on the locations and times of observations, the platform enables the visualization of mosquito population trends and ranges. It also serves as an educational resource, encouraging collaboration and data sharing. The data acquired and displayed on GMOD is freely available in multiple formats and can be accessed from any device with an internet connection.

**Results:**

Since its launch less than a year ago, GMOD has already proven its value. It has successfully integrated and processed large volumes of real-time data (~ 300,000 observations), offering valuable and actionable insights into mosquito species prevalence, abundance, and potential distributions, as well as engaging citizens in community-based surveillance programs.

**Conclusions:**

GMOD is a cloud-based platform that provides open access to mosquito vector data obtained from citizen science programs. Its user-friendly interface and data filters make it valuable for researchers, mosquito control personnel, and other stakeholders. With its expanding data resources and the potential for machine learning integration, GMOD is poised to support public health initiatives aimed at reducing the spread of mosquito-borne diseases in a cost-effective manner, particularly in regions where traditional surveillance methods are limited. GMOD is continually evolving, with ongoing development of powerful artificial intelligence algorithms to identify mosquito species and other features from submitted data. The future of citizen science holds great promise, and GMOD stands as an exciting initiative in this field.

**Supplementary Information:**

The online version contains supplementary material available at 10.1186/s12942-023-00350-7.

## Background

Mosquitoes and the diseases they transmit are a global health concern, resulting in greater than 17% of all infectious diseases [1]. Globally, mosquito-borne diseases infect about 700 million people every year, killing nearly one million humans annually [2] In particular, malaria accounts for at least 619,000 of these deaths each year [3–6]. Efforts to combat mosquitoes are becoming increasingly challenging, largely due to changes in climate, human expansion, and vast interconnected global travel and transport. Widely practiced methods for controlling mosquito-borne diseases target species of medical importance, requiring expert knowledge and a significant number of resources. Additionally, the risk of human error with manual identification is high, particularly when quantities of adult trap collections are processed en masse.

Fortunately, advances in mobile technology and greater affordability have provided everyday users with highly capable and portable handheld devices for a large variety of tasks. With over 6 billion users globally, smartphones are equipped with global position systems (GPS) and high-resolution cameras [7]. Combined with engaged community members and public science initiatives, smartphones are a significant force leading to the rapid rise in citizen science [8]. When applied to broader mosquito control questions, information ascertained from citizen science data is cost-efficient and scalable [9], providing a wealth of knowledge that can aid in reducing the global burden of mosquito-borne diseases.

Currently, there are several mobile phone applications that facilitate user-friendly citizen science participation. Collectively, vast amounts of data are available and provide an impressive assemblage, but several challenges have hindered translating this information into actionable change. Differences in interoperability, data formatting and consistency, and temporal and spatial coverages, discourage the standardization, merging, and analysis of data for sharing and use in future studies [10]. With respect to submissions specific to mosquitoes and their habitats, three global applications exist, creating four unique datasets: GLOBE Observer (Mosquito Habitat Mapper and Land Cover), Mosquito Alert, and iNaturalist [10–13]. Driven by the Global Mosquito Alert Consortium [10, 14, 15], we seek to bolster submissions from these applications and connect citizen scientist data globally through standard protocols pertaining to mosquitoes (especially those of medical importance), their habitats, and biting occurrences.

We created the Global Mosquito Observation Dashboard (GMOD, www.mosquitodashboard.org), a streamlined central repository where mosquito-specific data are harmonized according to open geospatial consortium (OGC) standards and presented in near real-time. GMOD fills the critical need for cross-platform integration of citizen science data for the detection and monitoring of invasive and vector mosquitoes across the globe—a problem of urgent concern in light of species such as *Anopheles stephensi*, the deadly urban malaria vector that has recently invaded the African continent (WHO 2022). Citizen scientists from around the globe submit their data to the three applications, where they are collected, processed, and provided to GMOD. These data are integrated through an intuitive, user-friendly interface with all four datasets available in summarized and raw formats, thus serving as a tool with immediate application for planning and initiating interventions aimed at reducing the burden of disease at local, regional, and global scales. Ultimately, by leveraging citizen science efforts from multiple platforms, GMOD increases access, interoperability, data sharing, and knowledge in the global fight against mosquito-borne diseases.

## Methods

In the spirit of the 50th anniversary of Earth Day, our objective was to follow the Global Earth Challenge’s call for the use of citizen science data that is findable, accessible, interoperable, and reusable (FAIR, globalearthchallenge.earthday.org) [16]. By leveraging open data created by the three citizen science programs, we created GMOD with the overall aim to increase awareness and develop consistent, real-time surveillance of mosquitoes globally with the final target of reducing the global burden of diseases. Partnering with GLOBE Observer, MosquitoAlert, and iNaturalist, our process began with the preparation, processing, and eventual integration of OCG-standard data.

### Data sources

#### GLOBE observer—mosquito habitat mapper and land cover

The Global Learning and Observations to Benefit the Environment (GLOBE) is an international science and education program sponsored by NASA and supported by NSF, NOAA, and the U.S. Department of State. Established in 1995, the GLOBE Program was originally implemented in K-12 classrooms. In 2016, the GLOBE Observer mobile application was released, enabling citizen scientists of all ages to participate in NASA science. GLOBE Observer is available for use in 15 languages across 127 countries. Within the application are two tools of primary interest for this research: the Mosquito Habitat Mapper (MHM) and Land Cover tool. MHM, launched in 2017, supports citizen science efforts to identify, locate, and eliminate breeding sites of medically important mosquitoes from *Aedes* (*Ae*.), *Anopheles* (*An.*), and *Culex* (*Cx.*) genera. In addition to mosquito specimens, MHM also enables users to document suitable habitats (e.g. standing water, tires, etc.) along with larvae and pupal observations (presence/absence and number). Users are encouraged to identify their mosquito submissions to genus, but are not required. The application guides users through a visual key to identifying mosquito larvae based on anatomical characteristics of the distal segment of the abdomen under magnification. Characteristics evaluated include the presence, absence, and appearance of the siphon, saddle, pecten, tufts, and comb scales.

Unique to MHM, users are prompted to report if they have the ability to mitigate mosquito habitats, by removing water and/or covering containers—a process called source reduction. This feature serves two beneficial purposes by (1) encouraging citizen scientists to be actively involved in community-based mosquito surveillance, and (2) actively reducing the burden of mosquito-borne diseases locally. The GLOBE Land Cover tool enhances characterization of the mosquito habitat observations collected with the MHM tool by allowing users to provide ecological conditions and additional photos of the environment around the targeted habitat. The interface has a built-in compass that orients users to provide six oriented photos for each location: north, south, east, west, up, and down.

#### Mosquito alert

Launched in 2014 and available in 18 languages, Mosquito Alert is managed by the following public research institutions: the Centre for Ecological Research and Forestry Applications (CREAF, https://www.creaf.cat/), Pompeau Fabra University (UPF, www.upf.edu), Catalan Institution for Research and Advanced Studies (ICREA, https://www.icrea.cat/), and the Blanes Center for Advanced Studies (CEAB-CSIC, http://www.ceab.csic.es/en/). Initially focused on controlling diseases spread by *Ae. albopictus* in Spain, the program has expanded to include citizen science collections targeting *Ae. aegypti*, *Ae. japonicus*, *Ae. koreicus*, and *Culex* spp. Mosquito Alert prompts users to send observations of adult mosquitoes, breeding habitats, and/or bites encountered. All adult mosquitoes submitted are validated by accompanying photographs by a team of entomologists.

#### iNaturalist

Launched in 2008, iNaturalist is a social network of citizen scientists connected via shared observations of biodiversity, extending to plants, animals, and fungi. However, insects comprise a significant portion of all submissions, amounting to ~ 25% of all observations. Of all insect submissions, mosquitoes total ~ 78,000 (as of mid-April, 2023), representing 407 species globally. iNaturalist relies on the method of identifying observations based on crowdsourcing. More specifically, observations classified as “Research Grade” maintain at least a 2/3 community member agreement for identifying species, or the next finest taxonomic level, if species level is not possible. Uniquely, iNaturalist also uses tens of millions of photos to train artificial intelligence (AI) models for identification suggestions on more than 38,000 taxa [17].

### Application development pipeline

#### Microsoft Azure data factory and storage

Data from each of the three sources are accessed via public URLs and stored in the Microsoft Azure Cloud. Using a scheduled Azure Data Factory pipeline, raw data is accessed daily from their respective source URLs and copied into an Azure Blob storage container called “Raw Data”. GLOBE MHM and Land Cover are accessed in a standard .csv format. Mosquito Alert is accessed in a flat-object .json format. iNaturalist, which uses unicode transformation format (UTF-16LE), is accessed as a lengthy multi-tiered nested-object .json format. The complexity of the iNaturalist data was so extensive that a special request to Microsoft was made to have it modify the way its Azure Data Factory copied .json data. Further, the data wrangling tool used to clean and standardize the iNaturalist data also required modification by its creators to enable such a large .json string to be ingested into its memory. Storage standards include that all data copied into the Azure Cloud has scheduled daily backups for 12 months being in a “hot” or quick-access state and after 18 months are put in “cold” or archived storage.

#### Trifacta—data standardization

Within the Azure Cloud, raw data is imported into a service called Trifacta, an application designed for data wrangling of large, raw datasets. Following the Open Geospatial Consortium (OGC) standard for data formatting, each data set is cleaned and restructured to match the OGC’s SensorThings format, enabling cross source data analysis [18]. Every change made to the data structure is tracked and recorded for documenting data provenance. Within Trifacta, raw data are transformed to the OGC standard with each transformation being documented in a “recipe.” Each recipe contains customized formatting of data, including text and number conversion, creation of new fields, calculations, etc. and can be found in Additional file 1. The resulting data set from each recipe is then exported in .json format to an Azure Blob storage container called “Derived Data.” Each recipe is scheduled to run daily after the raw data has been copied into the Azure cloud via Azure Data Factory. Since each dataset follows the OGC standard, all data is stored as nested-objects in .json format. To enable ingestion to the Esri ArcGIS® Online service, datasets are run through one more recipe which presents data as a flat .json data set.

### End user display customization and experience—Esri ArcGIS online applications

This project’s end user visual displays are created within the ArcGIS Online application interface. After Trifacta completes its daily transformation of the raw data, Esri’s ArcGIS Online web application (via ArcGIS® Notebooks) pulls each of the four flattened OGC-.json formatted data sets (GLOBE MHM, GLOBE Land Cover, MosquitoAlert, and iNaturalist) into the ArcGIS Online application interface and stores them as feature layers. Feature layers are then incorporated into the Web Map application element. This integration step is the first instance where all four data streams are displayed simultaneously. This map serves as the spatial representation of all submissions of mosquito, habitat, and land cover data and is the centerpiece to the overall dashboard visualization. In addition to the feature layers, each data set can be accessed via a RESTful API endpoint provided by Esri ArcGIS Online. An example flow of the steps performed by Python scripts in ArcGIS Notebooks can be found in Additional file 1.

#### Pop-ups

Within the web map application element, ArcGIS Online provides a variety of options to display and interact with data in the maps. Among these options is the pop-up configuration—a custom information box that “pops-up” on any given observation point when clicked by a user. With custom elements coded in the ArcGIS® Arcade expression language, pop-ups display alphanumeric text and media to aid in data exploration (Arcade code examples are found in Additional file 1).

#### Dashboards

ArcGIS Online includes ArcGIS® Dashboards, a dashboard creation tool that seamlessly allows users to build their display through a variety of build-in tools, including elements (e.g. headers, widgets, footers, graphs, data selectors, numeric indicators, etc.), layouts, and themes. Furthermore, within each tool are several additional customizable options for including media, dynamic text, external links, and other features.

#### Experience builder

The ArcGIS Dashboards tool is an excellent method for visually representing data dynamically. However, ArcGIS® Experience Builder provides a full suite of complementary tools that enables deeper data interaction and exploration, providing a true, custom website look and feel. The creation and success of GMOD relies on the premise of being free to everyone, intuitive, and most importantly, accessible. This extends to viewership on all platforms—desktop computer, mobile phone, and/or tablet screens. Given each of these screen types are variable in resolution and aspect ratios, separate dashboards were created for each (ArcGIS Dashboards creation tool provides options for different platforms). Experience Builder allows for all three screen types to be integrated, so that all platforms will share the same web address link. Another key feature of Experience Builder is the ability to link external websites as “buttons”. For use in GMOD, these clickable tabs provide an option to explore the raw and summarized data, view publications relevant to the dashboard, and an option for users to subscribe to our mailing list (connected through the included application ArcGIS® Survey123).

#### Hub

**Fig. 1:**
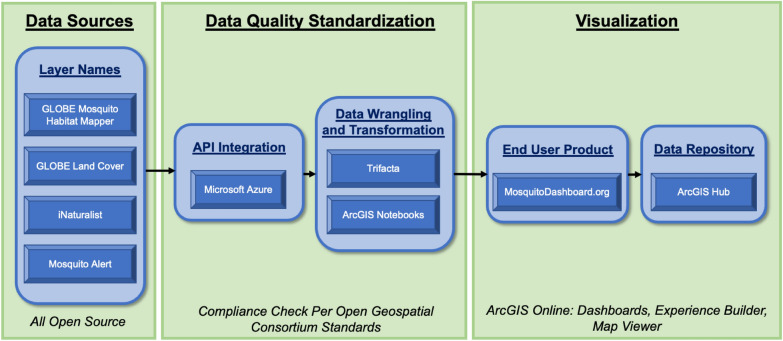
Flow diagram of data pipeline, from source to end-user dashboard product

The final key tool is ArcGIS® Hub℠, a cloud platform that facilitates rapid sharing and configuration of content. When ArcGIS® Open Data is enabled for an ArcGIS Online organization, Hub can be used to share an authoritative data repository that grants users full access to a variety of data formats, derived from the stored feature layers (.csv, .kml, .shp, GeoJSON, or file geodatabase). This feature is critical in our mission to fulfill data accessibility, transparency, and equity—all essential in promoting data sharing with those interested in using it. The data links to Hub are added via customizable “buttons”, created using Experience Builder. A summary of this entire workflow is available in Fig. 1.

## Results

### GMOD interface and features

The end-user product of GMOD is the culmination of data collected from citizen scientists (source), translated and checked for OGC standardization (Azure), converted to recipes and wrangled for uniformity (Trifacta), then displayed through a suite of tools and applications within Esri ArcGIS Online (Fig. 1). The majority of all features created in GMOD are designed for a customizable experience promoting user interaction and data exploration.

**Fig. 2:**
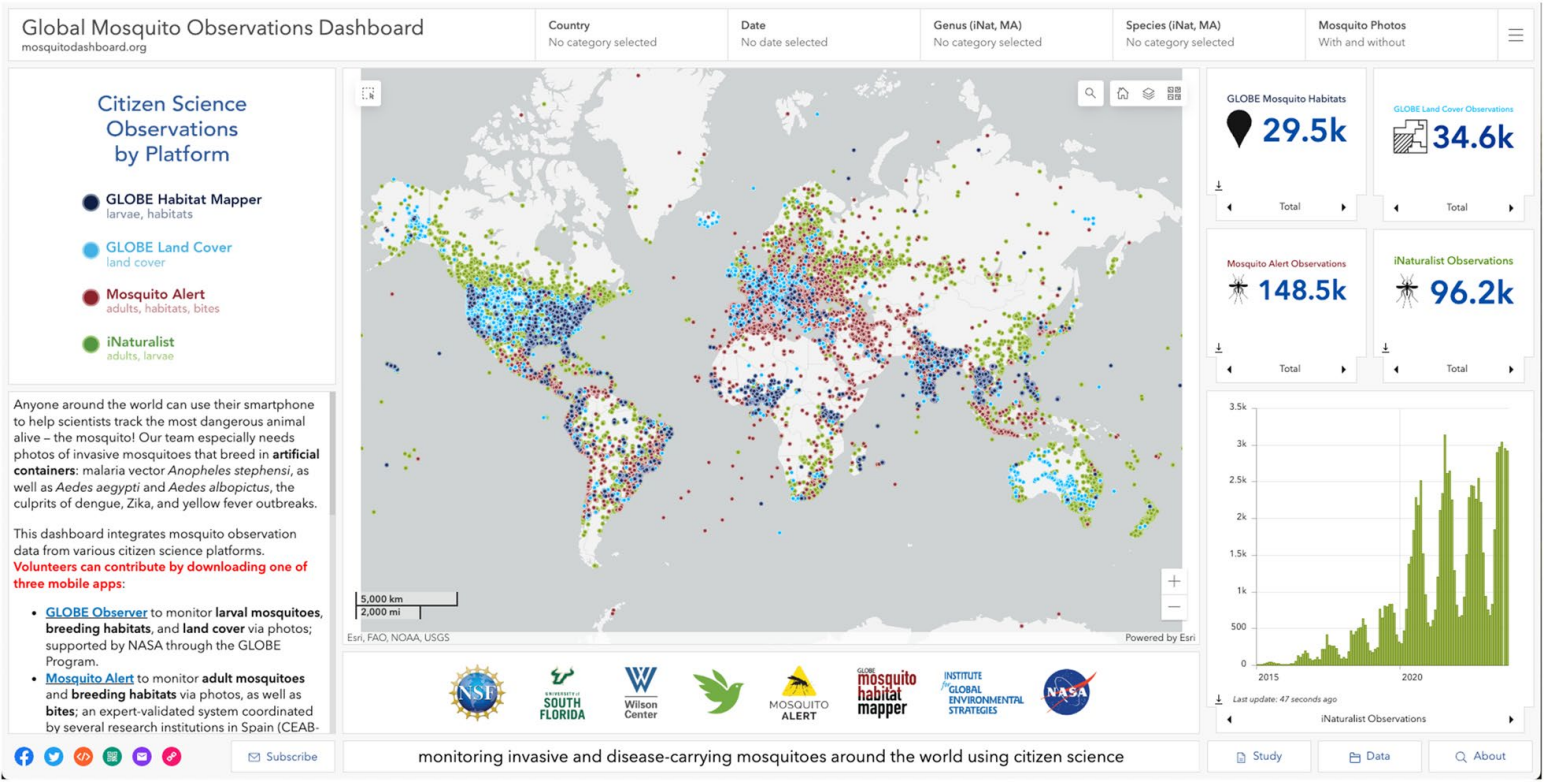
GMOD final end-user product (desktop version)

**Fig. 3:**
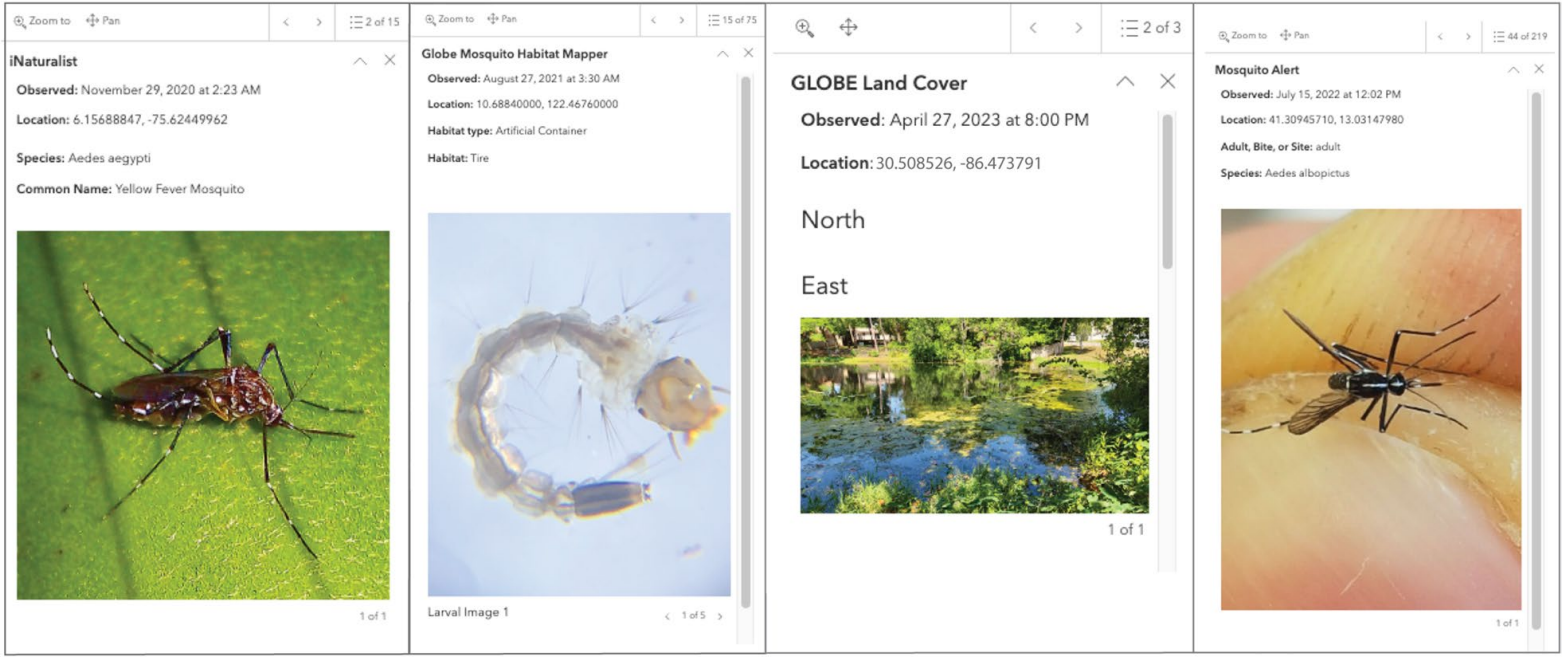
Summary of key information for each observation type, displayed within the configured pop-up feature

The far left side of GMOD contains the legend with colors corresponding to each of the data streams, as well as a description of GMOD and guide for navigating (Fig. 2). Highlighting the geospatial qualities of citizen scientist observations globally, GMOD is centered around the map element, displaying thousands of points from each of the four uniquely color-coded data streams. Clicking on any data point will open a pop-up feature, where users will see a summary of key information (Fig. 3). Each point will display the data source, observation date, spatial coordinates, and photo(s) (if provided). Mosquito observations will display species and common name, adult, bite, or size characteristics (iNaturalist submissions only), land cover (GLOBE Land Cover only), or habitat type (MHM submissions only). Outside of the map element, GMOD has a variety of different features users can explore. On the banner, users have the ability to filter submissions by country or countries (map layer will zoom to selection(s) and flash the border outline(s)), date range, mosquito genus and/or species (for iNaturalist and MosquitoAlert layers only), and by photo submission (contains or does not contain at least one photo). Users can also navigate to a specific location by typing in an address or place, toggle the various data layers on and off, and select from a variety of basemaps. If users would like to explore the original source applications, the far right of the header contains a toggle drop down selector that will open the respective website of interest. To the right of the map element are the data display features. Each data stream contains stacked widgets that display total observations and summary information of key elements for each respective data types (e.g. number of artificial containers, number of *Ae. aegypti*, bites, etc.). Below the widgets are interactive bar graphs of total submissions by year for each data stream, that update based on the selection(s) of features located in the banner. At the bottom of GMOD is the footer, which contains clickable features that all open new tabs to external websites or features. Users can find a variety of methods for sharing their content (URL links, social media, email, QR code) as well as a subscription button for updates and news emailed directly. On the far right of the footer are three additional buttons: studies and publications relevant to GMOD, data that users can download (raw or summary forms in CSV, KML, Shapefile, GeoJSON, or file geodatabase formats), and information about the original NSF funding source.

## Discussion

The creation of GMOD is a tool designed as a centralized digital repository for global mosquito and mosquito habitat data. Given the enormous potential in aiding ongoing scientific endeavors, our main goal was to leverage the vast resources of citizen scientists to enhance global mosquito surveillance and ultimately control efforts. The integration of hundreds of thousands of data points from our three partners, intuitive and interactive interface, and visually appealing aesthetics enables users to personalize their experience. This in turn, provides positive feedback that demonstrates value in their contributions to their communities, further encouraging and advocating for citizen science. Indeed, citizen scientists armed with smartphones can serve as extra sets of eyes to monitor mosquitoes, in locations and at a scale otherwise impossible via traditional mosquito trapping methods.

By contributing potentially actionable data on precisely where different mosquito species are found in their community, everyday citizens can help guide local mosquito programs in multiple ways. In the short term, such efforts can help inform mosquito surveillance and control efforts, especially during an epidemic. Currently, local transmission of malaria is occurring in the U.S. for the first time in two decades, and in three separate localities in Florida, Texas, and Maryland (7, 1, and 1 cases, respectively) [19]. In the light of these developments, GMOD has been highlighted as a public health tool for informing public awareness [19]. In the longer term, such data enables researchers to construct and validate mosquito habitat and risk models [20]. Third, such observations provide the ability to detect invasive species [21, 22], with particular relevance to vector species such as *An. stephensi* in Africa (WHO 2022). Fourth, by the public being aware of the problem and being engaged as part of the solution, they can help limit the spread of mosquito-borne diseases by eliminating standing water that serves as mosquito breeding habitats, as well as by practicing personal protection measures (e.g., using repellant with DEET).

GMOD values data accessibility and strongly encourages collaborations among the global community, reusing data, and sharing knowledge. Created by our partners and combined by GMOD, future analyses that are generated from these data can encompass any time duration (1 week or 1 year) or spatial domain (a single county or a continent). Since GMOD’s launch, several insights have been made; alterations and additions to research studies have been deduced to fill spatiotemporal “gaps” in observations, invasive species have been detected in new locations, and positive interactions with individual users continues to occur.

While the increased global accessibility and use of smartphones are on the rise, there are still large gaps in the reporting of mosquito data. These gaps exist in a variety of forms: by region of the world (Central and Northern Asia, and Central Africa vs. North America and Europe), socio-economic status (wealth disparities and affordability), and even within countries (urban vs. rural). This reporting bias results in point data correlated stronger with human population density (especially those with access to smartphones), rather than mosquito abundances.

The next phase for GMOD is perhaps the most consequential for mosquito-borne diseases—the integration of real-time mosquito identifications of each submission at the level of genus or species. Currently in development, preliminary efforts using machine learning algorithms have led to exciting results [23, 24]. For example, employing techniques such as the Mask Region-based Convolutional Neural Network highlighted key morphologic features (e.g. scutum, head, proboscis, wing patterns, etc.), and Class Activation Maps provided explainable AI insights for the adult classifications of our targeted invasive species *Ae. scapularis* that had > 90% accuracy [11]. These models can be used on larval and adult stages of dead or alive specimens, and all three mosquito datastreams are analyzed daily by our various AIs for the presence of *An. stephensi* specimens. Prior to this, our models had detected a suspected *An. stephensi* larval observation submitted via GLOBE in Madagascar (where this invasive species was not known to be present), which prompted targeted surveillance around the area [25] (mosquitoesInAfrica.org). This surveillance in turn yielded discovery of iNaturalist observations in Madagascar documenting a previously unknown phenomenon of mosquito-bite induced color change in chameleons [26], exemplifying how integrating multiple citizen science platforms together can yield unforeseen and serendipitous discoveries.

## Conclusions

The broader scientific community can benefit from the relatively untapped, massive resources that citizen science has to offer. Applications like GLOBE Observer, Mosquito Alert, and iNaturalist put the power in the hands of the public to help fight mosquito-borne diseases in their community. GMOD harnesses this data on a global scale, by collating and standardizing mosquito data into an easy to use, intuitive, real-time global observation dashboard. GMOD enhances the user experience by providing numerous tools to explore the data in unique ways. Most importantly, the data is free to use, easily accessible, and updated daily. The future of citizen science is promising and most importantly, pragmatic. Overall, such programs that leverage citizen science can empower communities and encourage even more advocacy and use, yielding benefits to the public and public health.

### Supplementary Information

**Additional file 1.** GMOD data layer API, ArcGIS Online Layer Details, Trifacta recipe for each platform, and ESRI scripting language examples.

## Data Availability

All data referenced in this manuscript are free and publicly available at www.mosquitodashboard.org. At the bottom right of the main page, click the ‘Data’ tab where a new window will open to download the raw data from each of the four platforms in numerous formats (.csv, .kml, ,shp, geojson, or file geodatabase). Individuals can also acquire this data from each of the respective partners at https://observer.globe.gov/get-data (GLOBE Observer), http://www.mosquitoalert.com/en/ (Mosquito Alert), or https://www.inaturalist.org/ (iNaturalist).

## Abbreviations

GMOD: Global Mosquito Observations Dashboard
*Ae.*: *Aedes*
*An.*: *Anopheles*
*Cx*: *Culex*
MBD: Mosquito-borne diseases
GPS: Global positioning system
OGC: Open geospatial consortium
GLOBE: Global Learning and Observations to Benefit the Environment
MHM: Mosquito Habitat Mapper
API: Application programming interface
UTF: Unicode transformation format
ESRI: Environmental Systems Research Institute
AI: Artificial intelligence
