# Supplementary material for: Global mosquito observations dashboard (GMOD): creating a user-friendly web interface fueled by citizen science to monitor invasive and vector mosquitoes
Source: Int J Health Geogr. 2023 Oct 28;22:28. doi: 10.1186/s12942-023-00350-7 (PMC10612222; doi:10.1186/s12942-023-00350-7)
Supplement: Supplementary file 1 — Additional file 1. GMOD data layer API, ArcGIS Online Layer Details, Trifacta recipe for each platform, and ESRI scripting language examples. [file 12942_2023_350_MOESM1_ESM.docx]

### **Additional file 1**

GMOD data layer API, ArcGIS Online Layer Details, and Trifacta recipe for each platform

**GLOBE Mosquito Habitat Mapper**:

*API:*

[https://api.globe.gov/search/v1/measurement/protocol/measureddate/?protocols=mosquito_habitat_mapper&startdate=2017-05-01&enddate=2030-01-01&geojson=FALSE&sample=FALSE](https://api.globe.gov/search/v1/measurement/protocol/measureddate/?protocols=mosquito_habitat_mapper&startdate=2017-05-01&)

*ArcGIS Online Layer Details:*

<https://www.arcgis.com/home/item.html?id=caeebdb90a6e4795a279166b28a8c36d>

*Trifacta Recipe:*

<https://drive.google.com/drive/folders/1P-EUp54nsEjo2TVPeHSg9U82r4wknLug?usp=sharing>

**GLOBE Land Cover**:

*API:*

<https://raw.githubusercontent.com/Piphi5/GLOBE-Clean-Datasets/main/General/Clean_LC.csv>

*ArcGIS Online Layer Details:*

<https://www.arcgis.com/home/item.html?id=432f90f584cf4598b669d0881aa3caf6>

*Trifacta Recipe:*

<https://drive.google.com/drive/folders/1A0SfApuhFCyQ4V_9wMxBZt56KfVsNw9R?usp=sharing>

**MosquitoAlert**:

*API (1 for each year):*

<http://webserver.mosquitoalert.com/fmapi/unep/dBEkteGZ78KDDJCI/all_reports2014.json>

<http://webserver.mosquitoalert.com/fmapi/unep/dBEkteGZ78KDDJCI/all_reports2015.json>

<http://webserver.mosquitoalert.com/fmapi/unep/dBEkteGZ78KDDJCI/all_reports2016.json>

<http://webserver.mosquitoalert.com/fmapi/unep/dBEkteGZ78KDDJCI/all_reports2017.json>

<http://webserver.mosquitoalert.com/fmapi/unep/dBEkteGZ78KDDJCI/all_reports2018.json>

<http://webserver.mosquitoalert.com/fmapi/unep/dBEkteGZ78KDDJCI/all_reports2019.json>

<http://webserver.mosquitoalert.com/fmapi/unep/dBEkteGZ78KDDJCI/all_reports2020.json>

<http://webserver.mosquitoalert.com/fmapi/unep/dBEkteGZ78KDDJCI/all_reports2021.json>

<http://webserver.mosquitoalert.com/fmapi/unep/dBEkteGZ78KDDJCI/all_reports2022.json>

*ArcGIS Online Layer Details:*

<https://www.arcgis.com/home/item.html?id=e550be48cc314395be870fd0c3a6906f>

*Trifacta Recipe:*

<https://drive.google.com/drive/folders/1_g-HoLGW4_-318vDce6szvWNm7gGCKCd?usp=sharing>

**iNaturalist:**

*API:*

<https://api.inaturalist.org/v1/observations?taxon_id=52134&page=1&per_page=200>

*ArcGIS Online Layer Details:*

<https://www.arcgis.com/home/item.html?id=bb853406b23d49d9ae2f97faceea4c41>

*Trifacta Recipe:*

<https://drive.google.com/drive/folders/1twT-rusIo6Uw4nZvSkMre3SUgqjRt43S?usp=sharing>

ESRI Scripting Language: Arcade - Useful script examples:

ArcGIS Arcade is a scripting language from Esri used to map values, create expressions for visualization, labeling, pop-ups, calculations, and aliases. A helpful start guide [can be found here](https://www.esri.com/about/newsroom/arcuser/arcade-4-steps/) and an excellent guide to functions [can be found here](https://developers.arcgis.com/arcade/function-reference/).

In creating GMOD, the Arcade language has been used almost exclusive within the context of customizing pop-ups. The following examples and references are pertaining to the most used and pertinent code when working with the dashboard. Most codes can be modified by evaluating existing expressions located within each layer’s pop-ups, and a vast amount of [documentation exists online](https://developers.arcgis.com/arcade/).

*Example 1: High-Resolution Image Resizing in Pop-Ups:*

Using the functions [Split](https://developers.arcgis.com/arcade/function-reference/text_functions/#split) and [Replace](https://developers.arcgis.com/arcade/function-reference/text_functions/#replace), the targeted field will be the field that houses your urls, then in the commas will be the delimiting character. In the example below, it is a semicolon. The second line will be important for displaying your split images. Type [0] for the first one and then 1, 2, 3, etc. for each subsequent one for as many as the max number of urls you need to display per feature layer field.

- Example script for parsing images and displaying in pop-ups:

Var splitURL = Split($feature.parameters, "mhm_AbdomenCloseupPhotoUrls': '")

var splitURL2 = splitURL[1]

var splitURL3 = Split(splitURL2, "', 'mhm_MeasurementElevation'")

var URLs= splitURL3[0]

//return URLs

var answer = split(URLs, "; ")

if (Count(answer)>5){

    return replace(answer[5],'square','original')

} else{

    return ''

}

*Example 2: Displaying images within pop-ups:*

In GMOD, we customized our data points to have clickable pop-ups with images of larval, habitat, and land cover features. In this example, we show an example script for integrating a user submitted larval photo.

Var splitURL = Split($feature.parameters, "Urls': '")

var splitURL2 = splitURL[1]

var splitURL3 = Split(splitURL2, "',")

var URLs= splitURL3[0]

//return URLs

var answer = split(URLs, "; ")

//console(answer[0],answer[1],answer[2],answer[3],answer[4],answer[5])

if (Count(answer)>0){

return replace(answer[0],'square','original')

} else{

return ''

}

*Example 3: Displaying latitude and longitude (for point data):*

function MetersToLatLon(mx, my) {

// Converts XY point from Spherical web Mercator EPSG:900913 to lat/lon in WGS84 Datum

// Fuente: http://www.maptiler.org/google-maps-coordinates-tile-bounds-projection/

var originShift = 2.0 * PI * 6378137.0 / 2.0;

var lon = (mx / originShift) * 180.0;

var lat = (my / originShift) * 180.0;

lat = 180.0 / PI * (2.0 * Atan( Exp( lat * PI / 180.0)) - PI / 2.0);

return [lat, lon];

}

var poly = Geometry($feature);

var result = "";

if (!IsEmpty(poly)) {

var pnt_centr = Centroid(poly);

var latlon = MetersToLatLon(pnt_centr.x, pnt_centr.y);

result = Round(latlon[0], 9) + "," + Round(latlon[1], 9);

} else {

result = "";

}

return result;

return result;

*Example 4: Designating mosquito habitat:*

Var splitURL = Split($feature.parameters, "'mhm_WaterSourceType': '")

var splitURL2 = splitURL[1]

var splitURL3 = Split(splitURL2, "'")

var URLs= splitURL3[0]

//return URLs

var answer = split(URLs, ",")

var replace1 = (replace(answer[0],'container: artificial', 'Artificial Container'))

var replace2 = (replace(replace1,'container: natural', 'Natural Container'))

var replace3 = (replace(replace2,'still: lake/pond/swamp', 'Lake/Pond/Swamp'))

var allReplaces = (replace(replace3,'flowing: still water found next to river or stream', 'Next to River/Stream'))

return allReplaces

Example flow of steps performed by Python scripts in ArcGIS Notebooks:

#Import necessary libraries

#Connect to the GIS

#Read primary feature layer ID and no geometry layer ID

#Read Azure connection string

#Read Azure container name

#Read list of columns that must be cast as strings

#Get the blob service client

#Get the container client

#List the blobs in the container

#Filter the blobs to get only the relevant names and save them to a list

#Define function to download the blobs

#Define function to process the blob json data and output a dictionary of spatial dataframes

#Define function to combine dictionary of spatial dataframes into common data frame and remove spaces from field names

#Define function to write records with no geometry to a feature layer

#Define progress indicator helper function

#Define retry helper function to try writing batches of edits to feature layer 3 times before failure

#Define helper function that returns retry function

#Define writing function that takes in write layer and feature set, batches the edits, and writes them to the feature layer using concurrent futures and above helper functions

#Define main function that calls all the above functions

#Call main function

**USF Specific Steps**

#Read feature layer ID for USF specific feature layer

#Read list of columns that must be cast as strings for USF specific feature layer

#List the blobs in the container

#Filter the blobs to get only the relevant names for USF layer and save them to a list

#Define a USF specific function to process the blob json data and output a dictionary of spatial dataframes, accommodating for differences between schema differences across years

#Define USF specific main function that calls above functions including USF specific processing function

#Call USF specific main function
